# 

**Published:** 1887-02

**Authors:** 


CONTENTS.

Vol. 34 No. 2.

| | PAGE. |
|---|---|
| Somnambulism | 23 |
| A Grander System of Human Life | 27 |
| What are we Drinking | 34 |
| Oxygen—Its Agency in Therapeutics III | 34 |
| Healthful Breathing | 42 |
| Fevers—Congestive and Inflammatory | 43 |
| Book Notices | 44 |
| Household | 45 |
| Miscellaneous | 47 |

INDEX TO ADVERTISEMENTS OF HALF A PAGE AND OVER.

| | PAGE. |
|---|---|
| Winchester's Hypophosphite | I |
| Lactated Food | II |
| Celerina | III |
| Lactopeptine | IV |
| Horsford's Acid Phosphate | V |
| Caulocorea | V |
| Crystalline Phosphate | VI |
| Bovinine | VII |
| Improved Fountain Syringe | VIII |
| Medicated Suppositories | XI |
| Dr. Carter Moffats' Ammoniaphone | X |
| Goodyear Rubber Co. | X |
| Perforated Paper | 3d page cover |
| Compound Oxygen | 4th " " |

